# Establishing Priorities for Improving Data Collection and Measurement of Mental Health and Well-Being of Adolescents With Special Educational Needs Within Nonmainstream Schools: Protocol for a Delphi Study

**DOI:** 10.2196/58610

**Published:** 2024-09-09

**Authors:** Mairi C Jeffery, Jessica Deighton, Suzet Tanya Lereya

**Affiliations:** 1 Evidence Based Practice Unit University College London and Anna Freud London United Kingdom

**Keywords:** special educational needs, special schools, alternative provision, mental health, well-being, school-based measurement

## Abstract

**Background:**

There are more than 1.5 million children and young people in England with special educational needs (SEN), with over 160,000 young people in the United Kingdom attending a special school or alternative provision (AP) setting. Young people with SEN have been found to be at risk for poorer mental health and well-being than non-SEN peers. However, there is a range of both school-related and research challenges associated with identifying difficulties in a timely manner.

**Objective:**

This Delphi study aims to determine a list of stakeholder priorities for improving school-based measurement of mental health and well-being among young people with SEN, at an aggregated level, within secondary special school or AP settings. A secondary objective is to inform the implementation of school-based well-being surveys, improve engagement in special schools or AP settings, and improve survey response rates among children and young people with SEN.

**Methods:**

A mixed methods Delphi study will be conducted, including a scoping review and preliminary focus groups with school staff members and researchers to establish key issues. This will be followed by a 2-round Delphi survey to determine a list of stakeholder priorities for improving the measurement of mental health and well-being at an aggregate level within special schools and AP settings. A final stakeholder workshop will be held to discuss the findings. A list of recommendations will be drafted as a report for special schools and AP settings.

**Results:**

The study has received ethical approval from the University College London Research Ethics Committee. The stage 1 scoping review has commenced. Recruitment for focus groups will begin in Autumn 2024. The first round of the Delphi survey will commence in early 2025, and the second round of the Delphi survey in the spring of 2025. The final workshop will commence in mid-2025 with final results expected in late 2025.

**Conclusions:**

There is a need for clear recommendations for special schools and AP settings on priorities for improving the measurement of mental health and well-being problems among young people with SEN. There is also a need for recommendations to researchers implementing school-based well-being surveys, including the #BeeWell program, to enable them to improve their engagement in special schools and AP settings and ensure surveys are accessible.

**International Registered Report Identifier (IRRID):**

PRR1-10.2196/58610

## Introduction

### Background

There are over 1.5 million children and young people in England with special educational needs (SEN) [1]. SEN covers a range of needs including speech, language, and communication needs; learning disabilities, autistic spectrum disorder, and attention-deficit/hyperactivity disorder; and social, emotional, and mental health needs [1].

There are high levels of co-occurrence between SEN conditions, which make it helpful to consider “special educational needs” as an umbrella term. For example, attention-deficit/hyperactivity disorder has been found to co-occur with conditions, including oppositional defiant disorder, conduct disorder, autism, anxiety, and coordination problems [2,3]. Likewise, autism has been found to co-occur with anxiety, disruptive, impulse-control, and conduct and depressive disorders [3].

Young people (adolescents aged 11-18 years) with SEN are at an increased risk of mental health problems [4], have lower levels of well-being than children without SEN [5,6], experience a more difficult transition to secondary school [7,8], and are more likely to experience bullying [9].

Many young people with SEN attend special schools or alternative provision (AP) settings, which are nonmainstream schools that cater to the different needs of young people. Special schools are those with specific educational provisions for young people with SEN or disabilities, while AP settings, which include pupil referral units (PRUs), are education settings for children and young people who are unable to attend a mainstream school for reasons such as being permanently excluded or having an illness that prevents them from being educated in a mainstream setting. In 2022-23, there were nearly 150,000 children with SEN in state-funded special schools and 4000 in nonmainstream special schools [10]. There were an additional 13,000 young people with SEN in state-funded AP settings and PRUs [1].

Most schools in the United Kingdom are taking steps to identify the mental health and well-being needs of their pupils. A report by the Department for Education found that 73% of special schools and 77% of AP settings carried out universal data collection on mental health [10]. However, large-scale school-based mental health and well-being surveys have been found to have barriers to implementation relating to competing school priorities, a lack of time and resources, and schools feeling overwhelmed by the number of competing surveys [11]. Recent research recommended that a program for school-based identification of mental health and well-being in mainstream primary school settings should (1) aim to identify children with the full range of severity of difficulties, (2) have opt-out parental consent, (3) educate staff members and children, and (4) provide a link between identified need and available support [12].

A school-based mental health and well-being survey, the #BeeWell program, found the administration of school-based surveys more challenging in special schools and AP settings. This was considered to be due to a high turnover of pupils, which made it difficult for schools to get necessary data-sharing agreements, consent, and resources in place in the time frame [13]. Likewise, due to smaller pupil numbers and lower response rates, which hindered the attainment of minimum data reporting thresholds, detailed aggregated well-being reports could not always be shared with special schools and APs [13]. This challenge is not unique to #BeeWell; other studies have had challenges with low numbers of young people with SEN in existing datasets [5] or did not collect data on SEN [14].

While there are general challenges with school-based measurement of mental health and well-being at an aggregate level, there are also challenges specific to identifying the mental health and well-being needs of children and young people with SEN. One such challenge is being able to distinguish between mental health difficulties and characteristics of special educational needs [15]. Hence, early warning signs can be missed, leading to late identification of issues [16]. Young people in AP settings may have their mental health needs viewed as behavioral issues, and there may be an overfocus on “challenging” behaviors rather than looking at the underlying emotional causes of behaviors [17,18].

Some children with SEN, particularly those with speech, language, and communication needs, may have difficulties in expressing their distress or describing negative events, such as bullying [19]. There may be difficulties relating to staff training and knowledge about mental health and well-being [15,18]. Young people with SEN may need help from school staff in completing surveys, which can be labor-intensive [20].

In addition, there is a lack of consensus on the most appropriate mental health and well-being measures for young people with SEN [21], particularly for those with intellectual or learning disabilities. A COSMIN (Consensus-Based Standards for the Selection of Health Measurement Instruments) systematic review of well-being measures for young people with intellectual disabilities found no measures that they could recommend for use [22]. However, more recently, an intellectual disability version of the Short Warwick–Edinburgh Mental Wellbeing Scale (ID-SWEMWBS) and KIDSCREEN-10 (ID-KIDSCREEN10) showed good psychometric properties [23].

### Aim and Objectives

This study aims to identify the challenges associated with conducting school-based surveys to measure the mental health and well-being of young people with SEN in secondary special schools and AP settings. It aims to reach a consensus among researchers and special school and AP staff on what the priorities are for improving data collection and measurement of mental health and well-being in these settings.

The findings will be used to draft guidelines for special schools and AP settings about improving the measurement of mental health and well-being issues among young people with SEN. These findings will also be used to make recommendations for school-based surveys to improve the engagement and response rates of special schools and AP settings in the future.

## Methods

### Design

This Delphi study will have a mixed methods design, including a scoping review and preliminary qualitative focus groups, with secondary special school and AP staff members and researchers in the field of mental health and SEN, to establish key issues relating to the measurement of mental health and well-being problems in SEN settings. This will be followed by a 2-round Delphi survey in the spring of 2025 to determine a list of the most important stakeholder priorities for improving the measurement of mental health and well-being at an aggregate level within these settings. A final follow-up workshop will be held in mid-2025 to discuss and reflect on the findings and provide additional information and context to the priority guidelines. It will be used to establish a list of the top 10 priorities if the final list from the Delphi survey rounds is long.

The Delphi method was chosen as it is useful for topics where there is a large amount of experiential knowledge among different expert groups and has frequently been used in mental health research [24-26]. The Delphi method was recently used in a similar study on stakeholder priorities for developing a program for mainstream school–based identification of mental health problems in mainstream primary schools [12].

It will also allow an agreement to be reached across different stakeholders (eg, special school and AP staff and researchers in different UK regions) about the priorities for addressing commonly faced issues in identifying mental health and well-being of young people with SEN at an aggregate level, which will be applicable to a wider range of special schools and AP settings. The Delphi method aims to make practical recommendations that can be implemented, and as the process is iterative, it allows participants to reflect on and revise previous responses, which is not possible in interviews or focus groups [24,27].

### Stakeholders and Setting

#### Participants and Recruitment: Focus Groups

Purposive sampling, which is a commonly used qualitative sampling technique to capture those who are especially knowledgeable about an area of interest (in this case, school-based measurement of mental health and well-being), will be used to recruit participants for the focus groups [28]. There will be 2-3 focus groups, each consisting of 5-8 participants: at least 1 focus group with school staff members and 1 focus group with researchers.

Participants for the “school staff” focus groups will be those working with adolescents with SEN in special schools or AP settings (including PRUs). These participants will be recruited from the sample of 38 special schools and AP settings who are currently taking part or have previously taken part in the #BeeWell study in Greater Manchester and Hampshire, Isle of Wight, Portsmouth, and Southampton. A smaller number of 5-10 schools will be contacted in the first instance, and further schools will be contacted based on responses and the need for additional participants. Targeted recruitment will be considered if there is a lack of representation from a particular school setting (eg, PRUs).

Initial contact to recruit school staff for focus groups will be made to school staff by an email sent by the #BeeWell project managers of Greater Manchester and Hampshire, Isle of Wight, Portsmouth, and Southampton. Individuals who are interested in participating will be encouraged to contact the researcher by email.

Participants for the “researchers” focus group will be those working in the field of SEN and mental health or on school-based mental health data collection (including researchers who have worked on the #BeeWell program but who are not associated with this study) and educational psychologists. These participants will be identified through purposive sampling, including from relevant university research groups such as UCL’s Centre for Research in Autism and Education or the University of Warwick’s Centre for Research in Intellectual and Developmental Disabilities.

Focus groups will aim for a maximum diversity of participants from different school settings, different regions, and different professional backgrounds.

#### Participants and Recruitment: Delphi Survey Rounds

To reach the minimum number of 50 responses needed in the round 2 survey, with anticipated dropout between rounds, a minimum of 100 participants will be recruited for the Delphi survey round 1. This will be approximately 50 school staff and 50 researchers or experts in mental health and SEN, including educational psychologists. There is no general consensus for the optimal number of participants to recruit in a Delphi study [29], but smaller sample sizes are preferred because the aim of the Delphi study is to establish a consensus between expert participants, rather than providing cross-sectional representation [30]. Furthermore, 1 review found that nearly 50% of Delphi studies had fewer than 40 experts [31].

School staff participants will be recruited from the special schools and AP settings who have been involved with the #BeeWell study in Greater Manchester and Hampshire, Isle of Wight, Portsmouth, and Southampton. Every special school and AP setting with previous #BeeWell involvement will be invited to complete the survey to capture a wide range of views. Participants will be those with knowledge of the school’s involvement with #BeeWell or with the implementation of school-based surveys on mental health and well-being. This study aims to have approximately 5-10 responses from each group of frontline teachers, special educational needs coordinators, head teachers, or other school leadership staff.

As in the focus groups, researcher participants for the Delphi survey will be identified through purposive sampling and snowball sampling of professionals in a research background, including university academic staff, who have published on mental health and SEN, and professionals in research policy or practice.

Participants will be recruited through email. Email addresses will be obtained from publicly available sources (eg, through author information on published journal papers or university staff lists). A general recruitment email will also be sent to research groups (eg, UCL’s Centre for Research in Autism and Education). Participants will be encouraged to forward the survey to colleagues with knowledge in the field of school-based measurement of mental health and well-being and SEN.

The perspectives of young people with SEN will also be incorporated as part of an associated study (UCL REC ethics approval project 26477/001). Young people with SEN, aged between 11 and 16 years, will be recruited from #BeeWell special schools and AP settings (more details are given in the “Phase 1: Delphi Survey Development” section below).

### Phase 1: Delphi Survey Development

Identifying the issues will be a 2-stage process. Stage 1 will involve a minimum of 2 focus groups. The first group will have between 5 and 8 school staff, and the second will involve 5-8 researchers or experts in mental health and SEN. If interested participants are unable to join the focus groups, they will be offered the opportunity to take part through individual interviews or a qualitative open-ended survey.

Focus groups will explore the barriers and facilitators to the measurement of mental health and well-being issues among children and young people with SEN in special schools or AP settings at an aggregate level. Focus groups will also incorporate questions about school-based surveys, such as the #BeeWell program, and improvements that could be made to the implementation of these surveys to improve special school and AP engagement and response rates.

Focus groups and interviews will be audio-recorded on an encrypted Dictaphone, and the audio recordings will be sent to an external transcription service for transcription.

Stage 2 will involve a scoping review to identify previously published literature on the school-based measurement of mental health and well-being problems among young people with SEN. The scoping review will explore the issues associated with school-based data collection and measurement, including the mental health and well-being measures that are validated for use in these settings. It aims to determine whether there are specific challenges referenced in the literature relating to the measurement of mental health and well-being problems within these settings (eg, staff time to implement surveys, barriers relating to parents opting out).

The perspectives of young people with SEN will also be incorporated during phase 1 Delphi survey development. Interviews with young people will be analyzed for any themes or nuances that have not been captured by the focus groups or the scoping review, such as young people’s feelings about their school collecting mental health and well-being data and issues surrounding data anonymity. As with the focus groups and scoping review, themes that emerge from interviews with young persons will be written into items for improvement of school-based measurement of mental health and well-being.

#### Data Analysis

Thematic analysis of focus group data will be carried out to identify statements for the Delphi survey. The process is based on a previous Delphi study [32]:

1. Audio recordings will be securely transferred to an external transcription service. Written transcriptions will be anonymized (removing names and identifying information) before qualitative data analysis is carried out.
2. Within the data, concepts generated from the discussion on issues will be extracted and grouped into categories. For example, themes may emerge about issues relating to “staff time,” “staff training,” “accessibility of mental health surveys,” “validated mental health and wellbeing measures,” or “communication barriers.” Themes might include those that are more general issues relating to special school and AP settings and themes that are more specific to research methods.
3. Issues related to school-based measurement of mental health and well-being among children and young people with SEN that emerged from the scoping review will also be categorized into items in the same manner as focus group discussions and sorted into the overall key themes.
4. Each key concept will be written as individual items for improvement, to create a provisional list of items for the Delphi survey. Each item will relate to only 1 idea and will be worded as a practical factor relating to an improvement that can be made in the measurement of mental health and well-being in school that can be rated on a Likert scale of importance from 1 (not important at all) to 9 (very important). For example, survey items may be “How important is it for adequate staff time to be available to assist young people with completing surveys?” “How important is it for accessible surveys for young people with SEN to be available?” “How important is it for school staff to have training on measuring the mental and wellbeing of young people with SEN?” and “How important is it for special schools or AP settings to have external support for implementing mental health and wellbeing surveys?”
5. The draft survey statements will be refined with input from an expert advisory panel with members drawn from both UCL and the University of Manchester.
6. A Delphi survey is then created by compiling a list of statements (grouped into themes) related to the research aims and objectives.

### Phase 2: e-Delphi Survey

#### Round 1

The first round of the Delphi survey will be piloted with 2-5 individuals from the #BeeWell advisory panel and colleagues at the UCL, to ensure clarity of statements and that there is no overlap between statements. The survey will be hosted on Qualtrics.

Invitations to complete the survey will be emailed to participants in early 2025, including those who participated in round 1 of focus groups, but also a wider number of participants from special schools and AP settings and researchers.

Participants will be emailed individually, and they will be reminded of the purpose of the study and asked to provide consent to take part in the survey, at the start. They will be informed about how to rate the statements and the importance of completing the Delphi survey in both rounds 1 and 2.

In the survey, participants will be asked for demographic data (age, gender, ethnicity, job role, and length of time in their field). They will then be asked to rate statements relating to issues on school-based data collection and measurement of mental health and well-being, on a Likert scale of importance from 1 (not important at all) to 9 (very important). A 9-point scale was chosen to be more sensitive to different scores, than a 5 or 7-point scale. As in a similar Delphi study, scores of 7-9 will be considered “important” issues, scores of 4-6 as “neither important nor unimportant,” and scores of 1-3 as “unimportant” issues [12].

The survey will include an open-textbox at the end for participants to recommend something that was not covered by the survey. Participants will have up to 3 weeks to complete the survey and will be sent up to 2 reminders to complete it.

#### Data Analysis

The results of the round 1 Delphi survey will be used to generate the survey for round 2. Statements will be considered to have reached a consensus if >70% of participants rated the statements unimportant (scores of 1-3) or important (scores of 7-9) and the statement’s IQR is less than or equal to 2, which has been done in a similar Delphi study [12]. Statements that reached consensus as “unimportant” will be dropped from the round 2 survey.

Any additional statements that were recommended by participants in the free textboxes in the round 1 survey will be reviewed by the researchers to ensure they are within the scope of the study, clearly worded, and are dissimilar to existing statements [25]; if they meet these criteria, they will be included in the next Delphi round.

#### Survey Round 2

Respondents of the round 2 survey who rate at least 50% of the statements will receive an invitation to the round 2 survey. Participants will receive feedback on the round 1 scores of all aggregated responses (ie, the aggregated scores from special school staff, AP and PRU school staff, researchers, and educational psychologists) as a median group-level score, alongside information about how they rated the statements.

In the round 2 survey, participants will be able to rerate the level of importance of the statements considering this feedback. As in round 1, participants will have 2-3 weeks to complete the survey and will be sent up to 2 reminder emails.

#### Data Analysis

As in round 1, statements will be deemed to have reached consensus if >70% of participants rated the statements unimportant (scores of 1-3) or as important (scores of 7-9) and the statement’s IQR is less than or equal to 2 [12].

All statements that achieve consensus as being “important” will be included in the final list of priorities for schools and researchers on improving school-based measurement of mental health and well-being.

### Final Follow-Up Workshop

A final follow-up web-based workshop will be held in early 2025 to discuss and reflect on the findings. The workshop will be used to establish a list of the top 10 priorities if the final list from the Delphi survey rounds is long. The workshop will aim to have 8-10 participants from a range of settings and UK regions recruited from those who completed both rounds of the Delphi surveys. The workshop will be recorded and transcribed to provide additional information and context to the priority guidelines.

### Ethical Considerations

This study will follow the Code of Conduct for Research of University College London. Ethics approval for this study was granted by the UCL Research Ethics Committee (REC; project 26477/002). This research project is allied to the #BeeWell program, which was reviewed and approved by The University of Manchester’s REC (reference 2021-11133-18179). The #BeeWell program introduced a youth-developed well-being survey to 187 secondary schools, including special schools and AP settings, across Greater Manchester in 2021. In Autumn 2023, #BeeWell began its rollout in Hampshire, the Isle of Wight, Portsmouth, and Southampton, with plans to extend further after 2024. There are 3 versions of the #BeeWell survey that include questions across the domains and drivers of well-being: a full version, a short version, and a widget symbol version, which was developed in consultation with young people in special school and AP settings.

#### Informed Consent

Participants will be provided with a detailed information sheet and privacy notice before participation in the research. For the focus groups, the consent form will be hosted on Microsoft Forms, the link to which will be sent along with the participant information sheet ahead of the focus groups. Participants will be informed that due to the nature of focus groups, full anonymity cannot be guaranteed for this stage of the Delphi study.

For the Delphi survey rounds, participant information and privacy notices will be explained within the web-based survey form, which will be hosted on Qualtrics. Consent for completing the surveys 1 and 2 will be obtained in the web-based surveys.

#### Privacy and Confidentiality

Participants will receive a privacy notice before involvement, which will state how their data will be collected, used, and stored. Names and identifying information of the participants, such as their school, will not be associated with their responses.

Due to the nature of focus groups, full confidentiality of responses cannot be guaranteed. However, participants will be informed about this in the participant information sheet and will be reminded at the start of the discussion to not discuss the focus group with others.

Individual schools or respondents will not be named in any outputs, and findings will be aggregated. It will not be possible to identify any individual survey responses within papers or reports.

#### Compensation Details

School staff who participate in the focus groups will receive a £25 (US $33) voucher for participation.

## Results

The stage 1 scoping review has commenced as of March 2024. The scoping review protocol is registered on the Open Science Framework [33].

Recruitment for focus groups will begin in Autumn 2024. The first round of the Delphi survey will commence in January 2025, and the second round of the Delphi survey in the spring of 2025. The final workshop will commence in mid-2025. Expected results will be published in late 2025.

## Discussion

### Anticipated Findings and Potential Impact

It is anticipated that this study will be able to identify a set of stakeholder agreed-upon priorities for special schools and AP settings and researchers, to be able to improve the measurement of mental health and well-being problems among young people with SEN. A report for special schools and AP settings will be written to provide feedback on the study’s findings related to priorities.

A set of guidance will also be written to make recommendations for the introduction or development of school-based mental health and well-being surveys, such as #BeeWell, to be more inclusive of special schools and AP settings and ensure a good response rate from young persons with SEN.

### Strengths and Limitations

The strength of this Delphi study is the range of stakeholders that will be included. It will include the perspectives of special school and AP staff who have direct experience with the challenges of trying to identify mental health and well-being problems among children and young people with SEN, along with other professionals working in the field of mental health and SEN, including educational psychologists. It will also incorporate the perspectives of young people with SEN themselves, who are being interviewed as part of an associated study.

As school staff will be recruited from specific schools who participated in the #BeeWell program, in 4 regions of the United Kingdom (Greater Manchester and Hampshire, the Isle of Wight, Portsmouth, and Southampton), it is acknowledged that results may be less applicable to other UK schools more widely. To minimize this risk, this study aims to recruit from different schools and include professionals from other areas who were not associated with the #BeeWell program.

Taking a multi-methods approach, including the qualitative focus groups in round 1, will generate a deeper understanding of some of the challenges that special schools and AP settings face when trying to understand the mental health and well-being problems faced by their students rather than just drawing on the literature.

### Conclusions

There is a strong need to identify some of the problems that special schools and AP settings face regarding measuring the mental health and well-being of adolescents with SEN. This Delphi study aims to establish a set of priority recommendations for improving school-based measurement of mental health and well-being, which have been agreed upon by stakeholders in special school and AP settings and researchers in mental health and SEN.

It also aims to make recommendations to researchers implementing school-based well-being surveys, including the #BeeWell program, to enable them to improve their engagement in special schools and AP settings, ensure surveys are accessible, and increase survey response rates of young people with SEN.

## Abbreviations

AP: alternative provision
COSMIN: Consensus-Based Standards for the Selection of Health Measurement Instruments
ID-KIDSCREEN10: intellectual disability version of KIDSCREEN10
ID-SWEMWBS: intellectual disability version of the Short Warwick–Edinburgh Mental Wellbeing Scale
PRU: pupil referral units
REC: Research Ethics Committee
SEN: special educational needs
